# WheatExp: an RNA-seq expression database for polyploid wheat

**DOI:** 10.1186/s12870-015-0692-1

**Published:** 2015-12-24

**Authors:** Stephen Pearce, Hans Vazquez-Gross, Sayer Y. Herin, David Hane, Yi Wang, Yong Q. Gu, Jorge Dubcovsky

**Affiliations:** Department of Plant Sciences, University of California, Davis, CA 95616 USA; USDA-Agriculture Research Service, Western Regional Research Center, Albany, CA 94710 USA; Howard Hughes Medical Institute, Chevy Chase, MD 20815 USA

**Keywords:** Expression, Wheat, RNA-seq, Polyploidy, Homoeologue-specific, WheatExp

## Abstract

**Background:**

For functional genomics studies, it is important to understand the dynamic expression profiles of transcribed genes in different tissues, stages of development and in response to environmental stimuli. The proliferation in the use of next-generation sequencing technologies by the plant research community has led to the accumulation of large volumes of expression data. However, analysis of these datasets is complicated by the frequent occurrence of polyploidy among economically-important crop species. In addition, processing and analyzing such large volumes of sequence data is a technical and time-consuming task, limiting their application in functional genomics studies, particularly for smaller laboratories which lack access to high-powered computing infrastructure. Wheat is a good example of a young polyploid species with three similar genomes (97 % identical among homoeologous genes), rapidly accumulating RNA-seq datasets and a large research community.

**Description:**

We present WheatExp, an expression database and visualization tool to analyze and compare homoeologue-specific transcript profiles across a broad range of tissues from different developmental stages in polyploid wheat. Beginning with publicly-available RNA-seq datasets, we developed a pipeline to distinguish between homoeologous transcripts from annotated genes in tetraploid and hexaploid wheat. Data from multiple studies is processed and compiled into a database which can be queried either by BLAST or by searching for a known gene of interest by name or functional domain. Expression data of multiple genes can be displayed side-by-side across all expression datasets providing immediate access to a comprehensive panel of expression data for specific subsets of wheat genes.

**Conclusions:**

The development of a publicly accessible expression database hosted on the GrainGenes website - http://wheat.pw.usda.gov/WheatExp/ - coupled with a simple and readily-comparable visualization tool will empower the wheat research community to use RNA-seq data and to perform functional analyses of target genes. The presented expression data is homoeologue-specific allowing for the analysis of relative contributions from each genome to the overall expression of a gene, a critical consideration for breeding applications. Our approach can be expanded to other polyploid species by adjusting sequence mapping parameters according to the specific divergence of their genomes.

## Background

Cereal crops provide a significant proportion of the calories consumed by humanity (http://faostat3.fao.org/) so maintaining and improving upon current production levels will be critical to provide food security for a growing world population. To meet this demand, continued and dedicated research efforts will be required to engineer solutions for the most pressing problems restricting agricultural production [1]. One important aspect of this research will be the identification and functional characterization of genes regulating the developmental stages most critical for determining yield and of genes which aid plant adaptation to a changing environment. Analyzing the dynamic expression profiles of each gene to describe their transcriptional regulation during the course of development, in different tissues and in response to specific environmental stimuli will be central to functional genetic studies.

In many economically-important crop species, such studies are complicated by polyploidy, the presence of two or more homoeologous genomes within a single nucleus. Polyploidy is widespread among plant species and is thought to aid the plant’s adaptation to diverse environmental conditions [2]. This increased adaptability is favored by the possibility of increased diversity in multimeric protein complexes and by global gene redundancy, which in some instances may be followed by gene divergence and sub- or neo-functionalization [2].

Wheat is one example of a recent allopolyploid species. The diploid species of the *Triticum*-*Aegilops* complex diverged from one another 3–5 Ma million years ago and are, on average, 97 % identical within the protein coding regions [3]. The hybridization of diploids *T. urartu* (AA genome) and a species of the *Sitopsis* group (BB genome) less than 500,000 years ago generated the tetraploid wheat species (AABB genomes) currently used predominantly for pasta. The hybridization of tetraploid wheat with *Aegilops tauschii* less than 10,000 years ago resulted in the hexaploid wheats (AABBDD genomes) currently used to make breads and pastries [4].

The complexity of the wheat genome, together with its economic importance and the existence of a large public research and breeding community make wheat an ideal target for the development of an expression database and the tools required to analyze and distinguish between homoeologues. This is now possible, owing to the recent release of a homoeologue-specific draft assembly of the wheat genome by the International Wheat Genome Sequencing Consortium (IWGSC) [3] and the publication of several RNA-seq expression datasets [5–10].

To assemble the wheat draft genome, individual chromosome arms were first separated according to size using flow cytometry. This allowed for the sequencing and subsequent assembly of each homoeologous chromosome arm separately. This was coupled with a broad effort to annotate gene-coding regions, using species-specific transcripts and prediction algorithms, as well as manual annotation. Annotated gene sets are regularly updated and released through the *Ensembl* genomics platform [11]. Thus, for the first time, comprehensive transcript profiling can be applied directly in hexaploid wheat to support functional genomics studies, including accurate separation of distinct homoeologous genes.

The recent, rapid advances in next generation sequencing technologies have proved transformative for wheat as for multiple other species, by providing the ability to sequence the entire transcriptomes of multiple biological samples at great depth, an approach known as RNA-seq [12]. Falling sequencing costs and streamlined library construction protocols have resulted in the proliferation of RNA-seq studies in diverse plant species [13]. Increasingly, large volumes of raw sequencing data generated from these studies are deposited in online repositories (e.g. Sequence Read Archive [14], Gene Expression Omnibus [15] or European Nucleotide Archive [16]). In addition to the specific research questions addressed by the authors of these studies, these datasets also represent a rich source of information for the wider research community. However, processing and analyzing such large volumes of data is a technically difficult, time-consuming task which requires bioinformatics expertize and access to computing clusters with high-performance infrastructure. This has limited the ability of small research laboratories and individual researchers to benefit from the wealth of information available in RNA-seq studies. To address this limitation and provide simple, free access to this data, we developed a pipeline to analyze transcriptomic data in polyploid genomes using wheat as a test case. Here we present WheatExp (http://wheat.pw.usda.gov/WheatExp/), an RNA-seq expression database and visualization tool that facilitates the analysis and comparison of homoeologous transcript profiles across a wide range of developmental and tissue samples in polyploid wheat.

## Construction and content

### Data sources and generation

All data contained within WheatExp is derived from RNA-seq reads deposited in online sequence repositories [14–16]. Currently, six complementary studies are included; a broad study of five different tissues across multiple timepoints [5], a study of seedling photomorphogenesis [6], a study of drought and heat stress in wheat seedlings [7], a study of wheat grain layers at a single timepoint [8], a senescing leaf timecourse [9] and a timecourse of different grain tissue layers during development [10] (Table 1). In combination, these datasets represent a diverse set of wheat expression data across multiple tissues, developmental stages and environmental treatments.

**Table 1:** RNA-seq datasets contained within WheatExp

Dataset	Wheat species	Tissues	Developmental stage/treatment	RNA-seq reads	% uniquely mapped reads	Data source	Reference
Wheat development timecourse	*T. aestivum* cv. Chinese Spring	Shoot, root, grain, spike and stem.	Three stages for each tissue	101bp Paired End (PE)	61.7 %	ENA: ERP004714	[5]
Photomorphogenesis	*T. monococcum* ssp. *monococcum* Acc. DV92	Whole seedlings	Etiolated and light-exposed seedlings.	50bp Single end (SE)	53.4 %	SRA: SRX283514	[6]
	*T. monococcum* ssp. *aegilopoides* Acc. G3116	Whole seedlings	Etiolated and light-exposed seedlings.	101bp PE	68.0 %	SRA: SRX257915	
Drought and heat stress	*T. aestivum* cv. TAM 107	Whole seedlings	Drought, heat and combined stress.	101bp PE	45.9 %	SRA: SRP045409	[7]
Grain layers	*T. aestivum* cv. Holdfast	Endosperm, inner pericarp, outer pericarp.	12 days after anthesis	50bp SE	31.4 %	ENA: ERP008767	[8]
Senescing leaf timecourse	*T. turgidum* ssp. *durum* L. cv. Kronos	Flag leaves	Heading date, 12 and 22 days after anthesis	50bp SE	33.9 %	GEO: GSE60635	[9]
Grain development timecourse	*T. aestivum* cv. Chinese Spring	Grain layers	10, 20 and 30 days after anthesis	101bp PE	56.1 %	ENA: ERP004505	[10]

*SRA* Short Read Archive, NCBI [14], *GEO* Gene Expression Omnibus, NCBI [15], *ENA* European Nucleotide Archive [16]

We designed a pipeline specific for polyploid wheat sequence data to analyze previously published RNA-seq datasets using a uniform set of tools and quality controls. The output of our pipeline is a set of expression values for all annotated wheat genes from the IWGSC project. Briefly, raw RNA-seq reads are first trimmed for quality and adapter contamination using two open-source packages, “*Sickle*” (https://github.com/ucdavis-bioinformatics/sickle) and “*Scythe*” (https://github.com/vsbuffalo/scythe), respectively, ensuring that only high-quality reads are considered when generating expression profiles. Trimmed reads are mapped to the full set of annotated homoeologue-specific wheat transcripts from the *Ensembl* genomics platform using BWA [17]. Uniquely-mapped reads are counted using “*Htseq-count*” [18] and then adjusted to derive RPKM/FPKM (Reads/Fragments per kilobase of transcript per million mapped reads) values for each gene based upon mapping rate, transcript length and library size. This normalization means that within a dataset, expression values are directly comparable across different tissues and developmental time points. Although the same reference was used for each dataset, comparisons across different datasets are less reliable because of differences in the number and length of sequencing reads between different datasets. Our mapping parameters are selected to report only those reads with a mapping quality (MAPQ) score of 40 from the Sequence Alignment/Map (SAM) file, a value which signifies that the read was mapped uniquely. Reads which map ambiguously, either to multiple homoeologues or to other identical sequences, have a lower associated MAPQ score, and are excluded in this step. Table 1 reports the % of reads mapped from each dataset after the application of this selection criterion. Across all six datasets, an average of 50.1 % of reads were mapped uniquely, resulting in homoeologue-specific expression data for each gene. In general, datasets with longer reads (e.g. 101 bp PE reads) resulted in a higher proportion of uniquely mapped reads than those comprised of shorter reads (e.g. 50 bp SE reads).

### Web implementation

The web interface was constructed using several different programming packages. The code base for the majority of the project is PHP (https://secure.php.net/) and JavaScript (https://www.javascript.com/). Relational database queries to the backend are performed with the PHP Data Object (PDO) module, allowing for secure queries. An additional advantage of using the PDO module is that the code is compatible with standard database engines such as MySQL, PostGreSQL and SQLite. In order to display dynamic graphs of the data, we implemented the HighCharts JavaScript library (https://github.com/highslide-software/highcharts.com). Specifically, this project uses a PHP module, which implements the HighCharts JavaScript library freely available on github (https://github.com/ghunti/HighchartsPHP). For dynamic text searches in portions of the website, the project implements Asynchronous JavaScript and XML (AJAX) technology using the package JQuery 1.11.3 (https://jquery.com/). Custom PHP and JavaScript code was written to develop a frontend website to enable BLAST [19] searches and to select multiple results for expression display. The site’s frontend was written in HTML and JavaScript with BLAST search [19] and AJAX gene identifier search forms allowing the user to select multiple results for expression display.

### Database implementation

The database implementation uses a flexible storage schema to house the data. The storage table has the following MySQL (https://www.mysql.com/) storage datatypes: study_id (varchar), seq_id (varchar), tissue (varchar), mean (float), se (float), se_low (float) and se_high (float). Binary search tree indices (BTREE) were implemented to increase the speed of queries using the study_id and seq_id columns.

### System architecture

The WheatExp tool is housed on the GrainGenes server at the following URL http://wheat.pw.usda.gov/WheatExp/. GrainGenes is an internationally recognized database for genomic and genetic resources in *Triticeae* and *Avena* species. The web and database is running on a ThinkMate ThinkTank IQ4 system with four Intel Xeon E7-4820 at 2.00 GHz with 52 GB of RAM. It is currently running Linux Kernel 3.13 distribution Ubuntu 14.04 long term support with PHP version 5.5.9 and MySQL version 5.5.43.

## Utility and Discussion

### Web interface

**Fig. 1:**
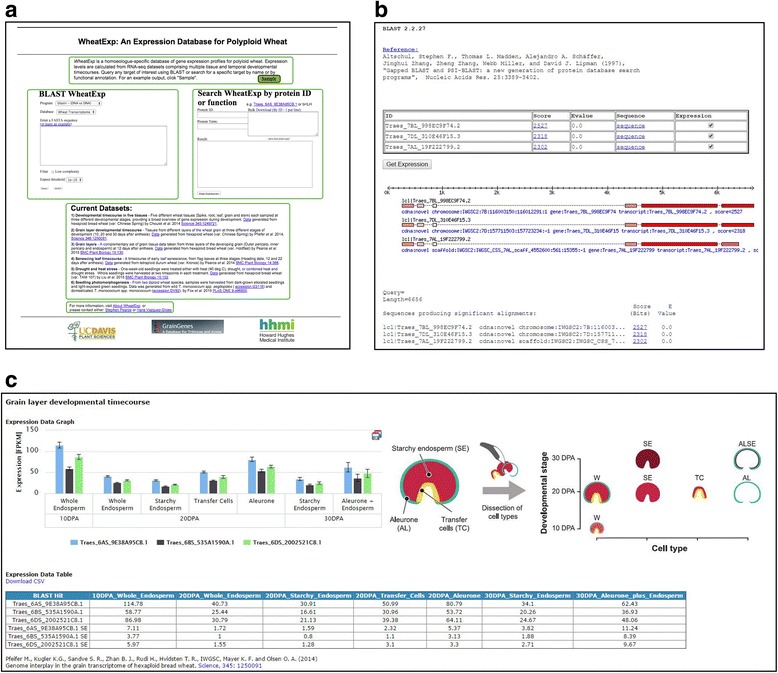
**a** Web interface of the WheatExp homepage. **b** BLAST results output page. **c** Screenshot of expression data visualization

The WheatExp homepage includes a brief description of the database and project design as well as details of all currently available datasets (Fig. 1a). Our data processing pipeline allows for the rapid incorporation of complementary RNA-seq expression datasets as they are published and we invite suggestions for the addition of new datasets from the user community. We anticipate regular expansions of the database to broaden the range of developmental and temporal expression profiles included. This approach will maximize the utility of the database for researchers studying diverse aspects of wheat development and ensures access to the most relevant high-quality expression datasets.

From this main hub (Fig. 1a), the database can be queried in one of two ways; either by entering the DNA or protein sequence of a gene of interest as a BLAST query, or by a text search for a known gene ID from the *Ensembl* genomics annotation platform [5] (e.g. Traes_6AS_9E38A95CB.1) or for an annotated functional term associated with the gene’s encoded protein (e.g. “bHLH” or “Cytochrome P450”). For BLAST searches, results are displayed on a new page and include details of each BLAST alignment, sequence and a link to the corresponding gene ID page on the external *Ensembl* genomics hub for simple cross-referencing (Fig. 1b). A maximum of six matched results may be selected for side-by-side display within the same graph to allow simple comparisons between multiple genes. While this feature was originally implemented to enable comparisons among wheat homoeologues, any set of up to six genes may be selected for comparison, regardless of their relationship.

Likewise, when browsing using the text search function, up to six genes can be selected for addition to the results list, which can subsequently be viewed side-by-side in the results window. For larger-scale analyses, tabular expression data for any number of genes can be downloaded by providing a list of *Ensembl* gene IDs of interest. The functional terms associated with each gene are obtained through standard gene annotation files in GFF3 format from the IWGSC which are stored within the database for text search function. We chose to adhere to the widely-used standard gene nomenclature format employed by the IWGSC and *Ensembl* genomics platform [5] and selected the set of annotated cDNA sequences from this platform as our mapping reference. External links to the annotated sequences for each gene are included in the results. This nomenclature format is increasingly becoming the standard for gene annotations within the plant research community, so our use of this reference will allow for the simple translation between projects and will maintain complementarity with the IWGSC project. This will facilitate comparative genomics studies with model plant species and other economically-important crops, such as rice, barley and maize, as the genomic resources contained within the *Ensembl* platform in each of these species improves. Additionally, comparisons can be made with more distantly related species to analyze functional gene divergence during the course of evolution.

Graphical expression profiles from all datasets are presented on a single results page, displaying mean RPKM/FPKM values +/− Standard Error Mean (SEM) (Fig. 1c). Graphs can be downloaded in one of four image formats and data is also presented in an accompanying table, which can be exported in ‘.csv’ format (Fig. 1c). Gene-level expression data can be downloaded separately, or in bulk as a single tabular file containing all data.

### Expression data

All expression profiles in WheatExp are generated from RNA-seq datasets. This approach has several advantages over existing expression studies derived from microarray data, which until recently, was the standard technology used for large-scale expression analysis (e.g. “Plant Expression database (PLEXdb)”, a database of microarray-based expression profiles in different plant species [20]). One of the advantages of RNA-seq is that it is an open platform that does not rely on predetermined sets of probes printed on a gene chip. In addition, this technology provides more reliable expression profiling across a broader dynamic range than is possible with microarrays.

**Fig. 2:**
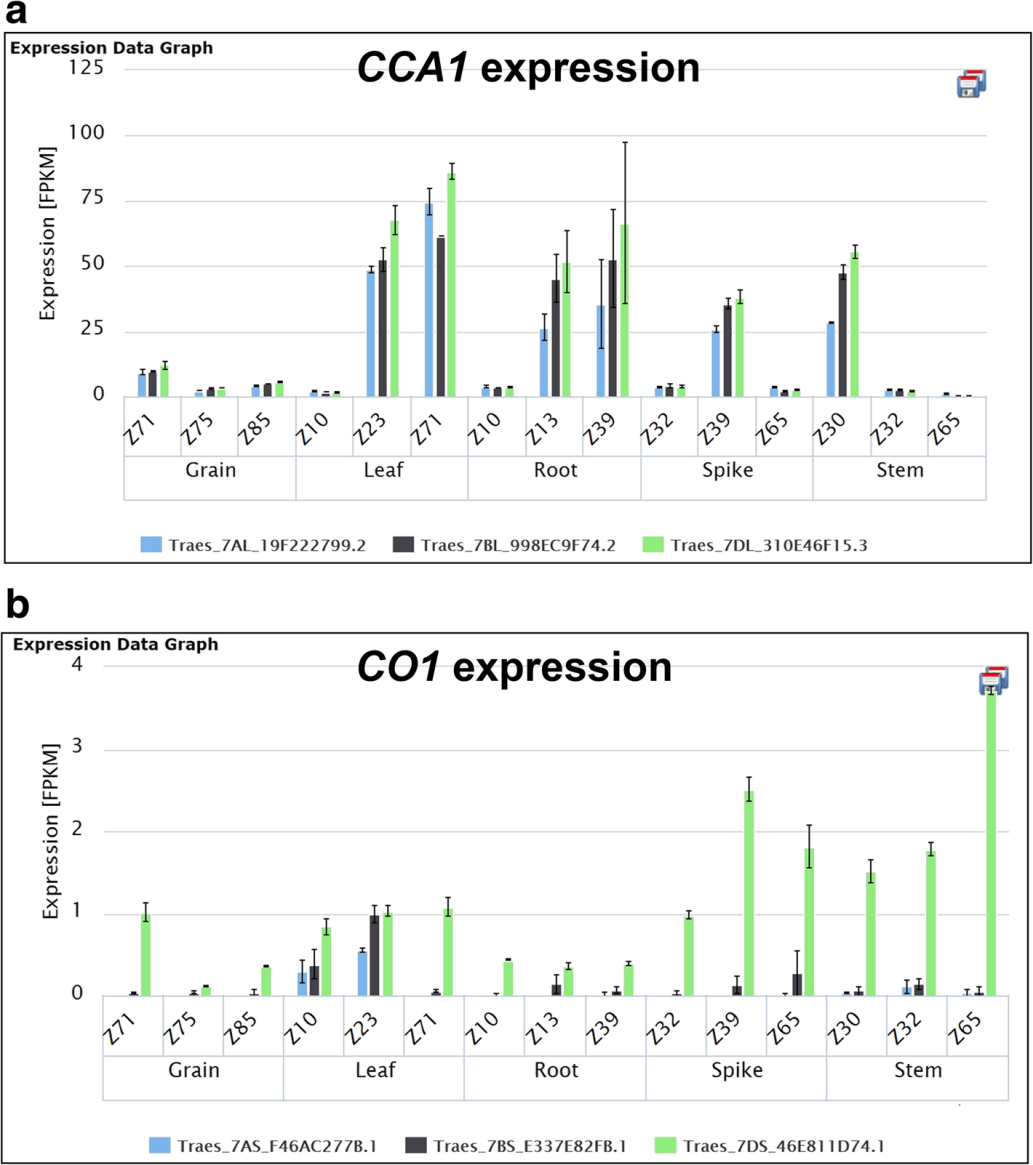
**a** *CIRCADIAN CLOCK ASSOCIATED1* expression evenly distributed between all three homoeologues. **b** *CONSTANS1* expression dominated by the *CO-D1* homeoologue during spike and stem development in hexaploid wheat

An important advantage of the application of RNA-seq data in polyploid species is that it facilitates the distinction among homoeologues and recently-diverged paralogous genes by allowing the application of stringent read mapping thresholds. Our selection of only uniquely-mapped reads has the dual benefit that the expression data are not only robust, but also homoeologue-specific, since the differences between these genomes (average 97 % identical) are distinguished by the selected mapping parameters. This is illustrated in Fig. 2 by two examples: *CIRCADIAN CLOCK ASSOCIATED1*, where the expression of the three homoeologous genes is approximately equal (Fig. 2a) and *CONSTANS1* where the D-genome homoeologue contributes the majority of transcripts to the overall expression (Fig. 2b).

### Simulated RNA-seq data

**Fig. 3:**
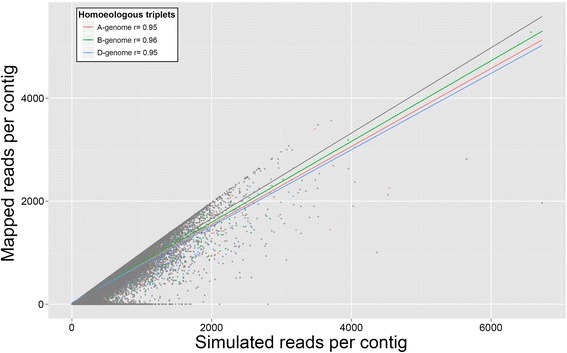
Scatter plot of synthetic read counts and observed mapping rates for each contig in the reference from a simulated RNA-seq experiment. Homoeologous triplets are highlighted in red (A-genome), green (B-genome) and blue (D-genome). All remaining contigs in the reference not classified as a homoeologous triplet are highlighted in grey

One drawback of using uniquely-mapped RNA-seq reads for expression analysis is that any read which maps equally well to identical regions in different genes is discarded, potentially resulting in an underestimation of the expression levels of highly similar genes [21]. To determine the extent of this effect in our database, we performed a simulated RNA-seq experiment. We generated 29.4 M synthetic 100bp paired-end reads with random expression levels and Illumina HiSeq2000 error profiles (‘ART’, mode art-illumina, default parameters except -m 500, −s 100 –ss HS20 [22]). All reads were processed using the same pipeline as for all biological RNA-seq data. By comparing the known number of simulated reads with the number of mapped reads, we can determine for each contig the proportion of reads discarded during mapping. Using a set of 3,476 homoeologous triplets (=10,428 genes) identified from a previous study [7], we mapped the subset of reads originating from each homoeologue to a reference comprised only of their genome of origin (i.e. A-genome reads were mapped to A-genome transcripts etc.). For the A, B and D genomes, an average of 98.6, 98.4, and 98.4 % of reads mapped uniquely to their transcripts of origin, respectively, demonstrating that only a small proportion of reads are discarded during mapping when their homoeologous genes are absent from the reference. When we repeated the mapping of all generated reads to the full reference, unique mapping rates were reduced to 82.4, 83.6 and 80.6 % for the A, B and D homoeologous triplets. In each case, this was a slightly lower unique mapping rate than for all remaining transcripts in our dataset (84.4 %). Despite this reduction in the mapping rate, we observed a high level of correlation between the number of generated reads and the observed mapped reads (r = 0.95, 0.96, 0.95 for A, B and D homoeologous triplets, Fig. 3). Therefore, while the estimated expression levels of homoeologous genes in our database are, on average, slightly reduced due to their sequence similarity, the reported expression remains closely correlated with the true expression level. Furthermore, this effect is approximately equal for transcripts originating from the three homoeologous wheat genomes (Fig. 3), demonstrating the absence of bias when comparing homoeologue-specific expression profiles for a gene of interest.

### Limitations

The main application of WheatExp is to compare the relative expression levels of the different homoeologues of a single gene across different tissues, developmental stages, environmental conditions and genetic backgrounds. For users interested in comparing the expression of different genes, we have included a statement on the website indicating that comparisons among genes are valid only when the genes being compared have the same number of homoeologues in the reference genome. Based upon results from our simulated RNA-seq experiment, genes where one homoeologue is absent from the reference will exhibit a higher proportion of uniquely-mapped reads and the expression levels of the two remaining homoeologues may also be inflated by the incorrect mapping of reads from the absent homoeologue. Additionally, no expression data will be reported for any genes which lack annotation within the current IWGSC release and any contig assemblies which are duplicated in the reference assembly will exhibit a reduced number of uniquely mapped reads. However, our project design allows for regular updates and refining of the mapping reference as this is expanded through the IWGSC project. As the mapping reference is improved we will re-map and re-process each dataset to generate updated expression sets using new versions of the reference, reducing the incidence and impact of such bias.

Our approach and data analysis pipeline can be applied to other polyploid species for which a homoeologue-specific genomic assembly is available to use as a reference. A critical parameter that must be considered in this application is the average level of identity among homoeologues, since this will affect the selection of the threshold for mapping uniquely mapped reads and thus the ability to discriminate between homoeologues.

## Conclusions

The increasing volume of expression data from RNA-seq studies represents a valuable source of information for the plant research community. We developed a pipeline tailored to polyploid wheat to rapidly process and analyze this data, and describe WheatExp, a database allowing the simple comparison of wheat homoeologue-specific sequences across a diverse set of temporal and spatial transcriptional profiles. Our database management is flexible, allowing for the incorporation of improvements in both the coverage of the wheat genomic reference and in the addition of complementary RNA-seq datasets released by third-party research groups. WheatExp provides simple, free access to a comprehensive array of expression data, empowering small labs and individual researchers to mine complex and valuable expression datasets.

## Availability and requirements

WheatExp is a free database and visualization tool open to all users with no login requirements and can be accessed at the following URL: http://wheat.pw.usda.gov/WheatExp/. The web tool is functional on all modern web browsing environments including Google Chrome, Mozilla Firefox and Safari.

## Availability of supporting data

All raw sequence data used to generate processed expression data for WheatExp is accessible from public sequence databases as described in Table 1. Processed counts and reference files are available for download through the WheatExp website.

## Abbreviations

AJAX: asynchronous javascript and xml
BTREE: binary search tree indices
ENA: European Nucleotide Archive
FPKM: fragments per kilobase of transcript per million reads mapped
GEO: gene expression omnibus
IWGSC: International wheat genome sequencing consortium
MAPQ: mapping quality
PDO: PHP Data Object
RPKM: reads per kilobase of transcript per million reads mapped
SAM: sequence alignment/map
SEM: standard error mean
SRA: sequence read archive
TILLING: targeted induced local lesions in genomes
